# Prevalence and Incidence of Atrial Fibrillation in Heart Failure with Mildly Reduced or Preserved Ejection Fraction: (Additive) Value of Implantable Loop Recorders

**DOI:** 10.3390/jcm12113682

**Published:** 2023-05-26

**Authors:** Thomas M. Gorter, Dirk J. van Veldhuisen, Bart A. Mulder, Vicente A. Artola Arita, Vanessa P. M. van Empel, Olivier C. Manintveld, Robert G. Tieleman, Alexander H. Maass, Kevin Vernooy, Isabelle C. van Gelder, Michiel Rienstra

**Affiliations:** 1 Department of Cardiology, University Medical Centre Groningen, University of Groningen, 9700 RB Groningen, The Netherlands; 2 Department of Cardiology, Cardiovascular Research Institute Maastricht (CARIM), Maastricht University Medical Centre, 6229 HX Maastricht, The Netherlands; 3 Department of Cardiology, Erasmus Medical Centre Rotterdam, 3015 GD Rotterdam, The Netherlands; 4 Department of Cardiology, Martini Hospital Groningen, 9728 NT Groningen, The Netherlands

**Keywords:** heart failure, preserved ejection fraction, mildly reduced ejection fraction, atrial fibrillation, implantable loop recording

## Abstract

Background: Atrial fibrillation (AF) is common in heart failure with mildly reduced or preserved ejection fraction (HFmrEF/HFpEF) and has a negative impact on outcome. Reliable data on prevalence, incidence, and detection of AF from contemporary, prospective HFmrEF/HFpEF studies are scarce. Methods: This was a prespecified sub-analysis from a prospective, multicenter study. Patients with HFmrEF/HFpEF underwent 12-lead electrocardiography (ECG), 24 h Holter monitoring, and received an implantable loop recorder (ILR) at the study start. During the 2 year follow-up, rhythm monitoring was performed via ILR, yearly ECG, and two yearly 24 h Holter monitors. Results: A total of 113 patients were included (mean age 73 ± 8 years, 75% HFpEF). At baseline, 70 patients (62%) had a diagnosis of AF: 21 paroxysmal, 18 persistent, and 31 permanent AF. At study start, 45 patients were in AF. Of the 43 patients without a history of AF, 19 developed incident AF during a median follow-up of 23 [15–25] months (44%; incidence rate 27.1 (95% confidence interval 16.3–42.4) per 100 person-years). Thus, after the 2-year follow-up, 89 patients (79%) had a diagnosis of AF. In 11/19 incident AF cases (i.e., 58%), AF was solely detected on the ILR. Yearly 12-lead ECG detected six incident AF cases and four of these cases were also detected on two yearly 24 h Holter monitors. Two incident AF cases were detected on an unplanned ECG/Holter. Conclusions: Atrial fibrillation is extremely common in heart failure with HFmrEF/HFpEF and may inform on symptom evaluation and treatment options. AF screening with an ILR had a much higher diagnostic yield than conventional modalities.

## 1. Introduction

Atrial fibrillation (AF) and heart failure (HF) with preserved ejection fraction (HFpEF) frequently co-exist and both increasing in prevalence [1]. Reported prevalence rates of AF in patients with HFpEF are highly variable and may depend on the study setting and study characteristics. For instance in recent HFpEF trials, the prevalence of AF ranges from 29 to 51% [2,3,4,5,6], whereas from community and registry data the prevalence ranges from 39 to 65% [7,8,9]. When present in HFpEF, AF is associated with lower exercise capacity, higher natriuretic peptide levels, and increased risk for all-cause mortality and hospitalization for HF [8,10,11,12,13].

The incidence of AF in patients with HFpEF is far less frequently investigated. In the Olmsted County population cohort, 32% of newly diagnosed HFpEF patients developed AF during a follow-up of 3.7 years [14]. In recent years, however, the criteria for the diagnosis of HFpEF have become far more stringent. Furthermore, in such a community-based study, many AF episodes may occur subclinical and remain undetected during follow-up, and thus incidence rates may be underestimated in such studies.

Since AF is highly prevalent in HFpEF and has a negative impact on outcome, the development of strategies to prevent the onset of AF in patients with HFpEF is important. Therefore, more insights into prevalent and incident AF in HFpEF from prospective studies with dedicated diagnostic tools are urgently needed.

In a prespecified sub-analysis from the ventricular tachyarrhythmia detection by Implantable loop recording in patients with heart failure and preserved ejection fraction (VIP-HF) study, we investigated the prevalence and incidence of AF in patients with HF with mildly reduced or preserved ejection fraction (HFmrEF/HFpEF) who all received an implantable loop recorder (ILR). In addition, we analyzed the diagnostic yield of an ILR for the detection of incident AF compared with yearly 12-lead ECG and two yearly 24 h Holter monitoring.

## 2. Methods

### 2.1. Study Design

The VIP-HF study was an investigator-initiated, prospective, multicenter, observational study conducted in The Netherlands between January 2015 and December 2019 [15]. In this study, patients with HFmrEF/HFpEF received an implantable loop recorder (ILR) at baseline that allowed for continuous rhythm monitoring during follow-up. The primary objective of the VIP-HF study was to study the incidence of sustained ventricular arrhythmias and these results were published in 2020 [15]. All patients provided written informed consent, and the study was approved by the local medical ethics committee. The study was in concordance with the principles outlined in the 1975 Declaration of Helsinki.

### 2.2. Study Population

The inclusion and exclusion criteria for the VIP-HF study were previously described [15]. In brief, patients were eligible for participation if they were >18 years old and had mild to moderate HF (New York Heart Association functional class II–III), and had a hospitalization or an emergency room visit for HF or were treated with diuretics for symptom relief in the past 12 months. Furthermore, patients were required to have LV ejection fraction (LVEF) >40% with additional echocardiographic evidence of myocardial functional and/or structural alterations: (1) septal or posterior wall thickness ≥11 mm, and/or (2) LV diastolic dysfunction (mean septal and lateral e′ < 9 cm/s, or E/e′ ≥ 13), and/or (3) left atrial (LA) dilatation (LA volume index ≥ 34 mL/m^2^). All patients were also required to have increased levels of N-terminal prohormone of B-type natriuretic peptide (NT-proBNP; >300 pg/mL for patients in sinus rhythm; >900 pg/mL for patients in AF). Patients with an internal cardiac defibrillator (ICD) or pacemaker or an indication for ICD/pacemaker therapy were excluded. Patients with a life expectancy of <1 year, as well as those with a myocardial infarction, percutaneous intervention, or coronary artery bypass graft in the past 3 months were also excluded.

### 2.3. Definition of Atrial Fibrillation

The prevalence of AF at baseline was defined as a previous diagnosis of atrial fibrillation or flutter according to the European Society of Cardiology guidelines. According to these guidelines, paroxysmal AF was defined as AF that terminates spontaneously or with intervention within 7 days of onset; persistent AF was defined as AF that is continuously sustained beyond 7 days, including episodes terminated by cardioversion (drugs or electrical cardioversion) after ≥7 days; and permanent AF was defined as AF that is accepted by the patient and physician, and no further attempts to restore/maintain sinus rhythm are undertaken [16].

### 2.4. Study Procedures

Echocardiography was performed at baseline and echocardiographic measurements were performed according to the current recommendations [17], and they include LVEF, LV mass index, pulsed-wave tissue Doppler velocities (e′) at the lateral and septal mitral annulus, E/e′ ratio, left atrial volume index, tricuspid annular plane systolic excursion, and tissue Doppler imaging of the right ventricular (RV) lateral wall (RV s′). At baseline, cardiac magnetic resonance (CMR) imaging was performed for the acquisition of cardiac volumes and functional parameters. All CMR studies were performed using a 1.5 Tesla scanner (Philips, Amsterdam, The Netherlands and Siemens, Erlangen, Germany). Electrocardiogram-triggered cine loop images were obtained during breath hold at end-expiration using a retrospectively gated cine steady-state, free-precession sequence. Cine loop images were analyzed off-line using dedicated software (QMass 7.6 and 8.1, QStrain 2.0, Medis, Leiden, The Netherlands). End-diastolic volumes and end-systolic volumes were automatically calculated by the summation of slices multiplied by slice thickness method. Volumetric measurements were indexed for body surface area according to the Dubois formula. RV-pulmonary artery coupling was calculated as RV stroke volume divided by RV end-systolic volume. Using the long-axis slices, LA and right atrial (RA) volumes were measured by tracing the area and length of both atria in end-systole and end-diastole. Atrial volume was approximated using the area-length method. LA and RA emptying fraction were calculated using the standard formula. Strain was measured as peak deformation of the myocardium from baseline to maximum length. LV and RV global longitudinal strain, LV circumferential strain, and LA and RA reservoir, conduit, and booster strain were measured. At baseline, all patients underwent blood sampling via venipuncture and EDTA anticoagulated plasma was obtained. NT-proBNP was measured using the Roche Modular system (Roche, Mannheim, Germany).

#### 2.4.1. Implantable Loop Recorder

All patients received an Abbott^®^ (Chicago, IL, USA) implantable loop recorder (ILR), type Confirm^®^ model DM2102 or Confirm Rx^®^ model DM3500. The algorithm used for rhythm monitoring was the same for each ILR type and was described previously [15]. Atrial fibrillation was programmed with lower priority. To minimize the risk of ILR noise capture, the sensitivity of the ILR was tested at every follow-up visit using the R-wave amplitude, and the ILR was adjusted accordingly.

#### 2.4.2. Rhythm Monitoring

The 12-lead ECG was performed at baseline for 10 s before implant of the ILR, and at 1- and 2-year follow-up. AF presence was defined as AF rhythm on a single 12-lead ECG recording.

Three-channel Holter monitoring during 24 h was performed at baseline before implant of the ILR, and at 2-year follow-up. AF presence was defined as the presence of at least one AF episode lasting >30 s on a 24 h Holter recording.

After ILR implantation, all patients were seen for ILR device interrogation at least every 6 months until the end-of-study visit at the two-year follow-up. The end-of-study visit was postponed for some patients due to the COVID-19 pandemic. For these patients the total follow-up was longer than two years, and the longest follow-up period was 31 months. All single-lead, bipolar surface ECG recordings that were stored by the ILR were assessed by a blinded endpoint adjudication committee that consisted of two experienced electrophysiologists (A.H.M. and R.G.T.). In case of disagreement between the two observers, a third independent electrophysiologist (M.R.) was consulted. The independent adjudication committee also confirmed (or rejected) the definite diagnosis of AF, which was defined as the presence of at least one AF episode lasting >30 s. The detection of new AF on the ILR was communicated to the treating physician, and further management was left to the discretion of the treating physician.

### 2.5. Statistical Analysis

Data are presented as mean  ±  standard deviation or median with interquartile ranges, depending on the distribution. Categorical data are presented as the number with percentages. Differences in continuous variables between groups were analyzed using the independent samples *t*-test or Wilcoxon rank test, depending on the distribution. Differences in categorical variables between groups were analyzed using the Chi-squared test or Fisher’s exact test. The incidence rates of AF with corresponding confidence intervals was calculated using the package epiR in R version 4.0.5 [18]. Associations between baseline characteristics and incident AF were assessed using a Cox proportional hazard regression model. Statistical analyses were performed using SPSS (Version 23; Chicago, IL, USA). Statistical significance was considered achieved at a *p*-value < 0.05.

## 3. Results

In total, 113 patients were included in the VIP-HF study. At baseline, 70 patients (62%) had a history of AF. Of these, 21 (30%) had paroxysmal AF, 18 (26%) had persistent AF, and 31 (44%) had permanent AF. The baseline characteristics of patients with and without a history of AF are depicted in Table 1.

### 3.1. Clinical Associations with Prevalent Atrial Fibrillation

Patients with AF were older, had larger left atria, and lower left and right atrial reservoir strain and emptying fraction. Patients with AF also had worse RV systolic function and more RV-pulmonary artery uncoupling. As expected, patients with AF also had higher NT-proBNP serum concentrations. Patients without a history of AF had lower LV diastolic tissue velocities and higher LV mass.

Of the 70 patients with a history of AF, 25 (36%) were in sinus rhythm at baseline and 45 (64%) were in AF. The characteristics between AF patients who had sinus rhythm versus AF rhythm at baseline are depicted in Supplementary Table S1. Patients who were in AF at baseline, were older, had lower LVEF and LV global longitudinal strain, more RV systolic dysfunction and more RV-pulmonary artery uncoupling, higher LA volume, and lower left and right atrial emptying fraction and reservoir strain compared with AF patients with a history of AF but who were in sinus rhythm at baseline (Supplementary Table S1).

Furthermore, when analyzing only the patients who were in sinus rhythm, the patients who were in sinus rhythm but had a history of AF were more often women, had lower LV mass index, higher RV end-systolic and end-diastolic volumes, and more RV uncoupling compared with patients in sinus rhythm without a history of AF (Supplemental Table S1).

### 3.2. Incident Atrial Fibrillation and Diagnostic Yield for Atrial Fibrillation Detection

**Figure 1:**
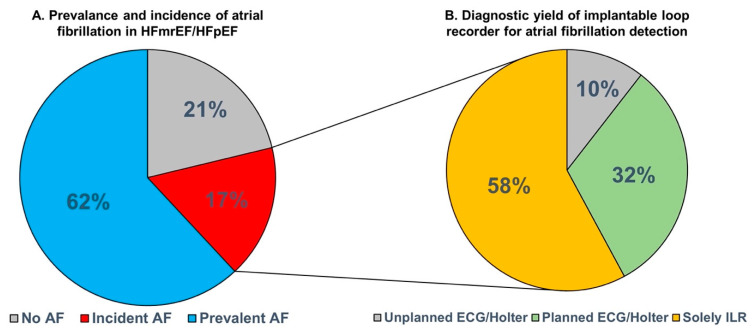
Atrial fibrillation in patients with heart failure with mildly reduced or preserved ejection fraction. (**A**): the prevalence and incidence of atrial fibrillation. (**B**): the detection of incident atrial fibrillation. AF: atrial fibrillation; ECG: electrocardiography; HFmrEF: heart failure with mildly reduced ejection fraction; HFpEF: heart failure with preserved ejection fraction; ILR: implantable loop recorder.

Of the 43 patients without a diagnosis of AF at baseline, 19 patients developed incident AF detected on the ILR during a median follow-up of 23 [15,16,17,18,19,20,21,22,23,24,25] months (44%; incidence rate 27.1 (95% CI 16.3–42.4) per 100 person-years), as seen in Figure 1A. The longest single AF episodes recorded among the study participants ranged from 1:03 min to 102 days. AF burden ranged from <1 to 61%.

Eleven (58%) of all incident AF cases were solely detected on the ILR (Figure 1B). On the contrary, yearly 12-lead ECG detected six (32%) incident AF cases and in four of these cases, AF was also detected in the two yearly Holter monitors. In two patients (10%) with incident AF, AF was detected on an unplanned 12-lead ECG or unplanned 24 h Holter within the 2-year follow-up period. The median interval between first detection of AF on the ILR and the subsequent detection on any (un)planned ECG or 24 h Holter was 4.3 [0.2–12.5] months. Supplementary Figures S1 and S2 depict the AF free survival based on ILR-detected AF and AF detected on any (un)planned ECG or 24 h Holter, respectively.

Table 2 and Table 3 show the Cox proportional regression model for the clinical associations with incident AF. LA volume index, assessed on echocardiography and cardiac MRI, was associated with incident AF. The associations remained significant, after adjustment for age and sex (*p* < 0.05 for LA volume index on echocardiography and LA end-diastolic and end-systolic volume index on CMR).

Nine patients (47%) with newly detected AF during follow-up were subsequently treated with direct oral anticoagulation or with vitamin K antagonist, as per discretion of the treating physician. In all eight patients in whom AF was also detected on any (un)planned ECG or 24 h Holter monitoring, anticoagulation was subsequently initiated. One patient who had AF solely detected by the ILR was also treated with anticoagulation. This patient had 9% AF burden with the longest single AF episode lasting >17 h. In the other 10 patients in whom AF was solely detected on the ILR, oral anticoagulation was not initiated before the end of the study. In one patient, AF was postmortem detected on the ILR with the first episode occurring three days before death. In the other nine patients, AF burden was relatively low (i.e., ≤2%) and the longest single episodes all lasted <12 h. None of the patients suffered from any ischemic or hemorrhagic stroke within the two-year follow-up period. By follow-up, three patients with incident AF had died. In the group of patients without incident AF, there were also three patients who died by the two-year follow-up.

None of the patients with incident AF underwent AF ablation before the end of the study. There was also no initiation of class I, III, or IV antiarrhythmic drugs before the end of the study in patients with newly detected AF. One patient was implanted with a leadless VVI pacemaker because of a bradycardia–tachycardia form of sinus node dysfunction before the end of the study.

## 4. Discussion

The present study had several interesting findings: First, approximately two-thirds of patients with HFmrEF and HFpEF had prevalent AF, which was associated with profound atrial myopathy and with more impairment of RV function and morphology. Interestingly, the latter association was in part irrespective of AF rhythm. Second, of the patients without AF at baseline, almost half of them developed AF during a 2-year follow-up period. After the 2-year follow-up, 79% of all patients had a diagnosis of AF and increased left atrial volume was independently associated with incident AF. Third, the majority of incident AF cases was solely detected on the ILR and were not detected on conventional 12-lead ECGs and 24 h Holter monitoring.

The AF prevalence of 62% in these patients with HFmrEF and HFpEF is relatively high compared with data from previous community-based studies and registries, in which the prevalence of AF ranges from 39 to 65% [7,8,9]. The higher prevalence may be due to the application of relative stringent inclusion criteria in the present study, including the requirement of a prior hospitalization for HF and/or symptom relief with diuretics in combination with an increased NT-proBNP. These criteria potentially lead to the inclusion of a more advanced form of HFpEF. On the other hand, the finding that patients with AF were older, had higher NT-proBNP levels, more severe atrial myopathy, and more RV systolic dysfunction is in line with several previous studies in patients with HFpEF [8,9,10,11,14,19,20].

In a previous community-based study in the Olmsted County population, Zakeri et al. showed that 32% of patients with HFpEF were subsequently diagnosed with new onset of AF [14]. This corresponds with an incident rate of 6.9 per 100 person-years, which is considerably lower than the present incident rate of 27 per 100 person-years. While the baseline characteristics in both studies are relatively comparable, the higher incident rate in our study is likely caused by the study setting and by the type of AF screening, namely continuous monitoring by the ILR versus ‘any documented AF on a clinically indicated ECG’ in the study by Zakeri et al. [14]. Indeed, the latter study was a retrospective analysis of a community-based cohort of individuals who were identified up to 40 years ago (i.e., between 1983 and 2010). Moreover, all new AF episodes occurred within a 2-year period in our study, whereas the median time to develop AF was 3.1 years in the Olmsted County cohort. Not surprisingly, continuous monitoring with an ILR leads to a higher detection rate of AF because many AF episodes occur sub clinically [21]. However, the diagnostic yield was considerably higher than routine (planned) rhythm monitoring using 12-lead ECG with a one-year interval and 24 h Holter monitoring with a two-year interval. As previously shown in an analysis from the LOOP study among individuals with increased CHA_2_DS_2_-VASc score but who were free of HF and AF, the diagnostic yield of screening for AF indeed depends on the duration, dispersion, and amount of AF screenings [22]. However as illustrated in the LOOP study, even twice-daily 30 s ECGs during 14 days or 30-day continuous Holter monitoring had a relatively low diagnostic yield (i.e., 11 and 34%, respectively), compared with the ILR [22]. Both the VIP-HF study and the LOOP study used an ILR for AF screening. To date, several other, less invasive systems and wearables are also available for AF screening, such as smartwatches, wearable belts, and patient-initiated ECG rhythm strips using a smartphone or connectable device. However, the sensitivity and specificity for AF detection may vary considerably between these AF screening tools [16]. A previous, smaller study also used an ILR to screen for (subclinical) arrhythmias in 30 stable outpatients with chronic HF, of which 20% had HFpEF. This study showed that eight patients (27%) developed AF during a median follow-up of 12 months. AF was detected in only one patient with HFpEF [23]. All newly detected episodes in that study led to the initiation of oral anticoagulation. In the present study, anticoagulation was initiated in 9 out of 19 patients with incident AF, albeit in the vast majority of these patients, clinical AF was confirmed on conventional ECG and/or Holter monitoring. It is currently unknown whether patients with device-detected, subclinical AF should be treated with oral anticoagulation. In a previous analysis from ASSERT, it was demonstrated that the incidence of device-detected, subclinical AF episodes lasting >24 h was associated with increased risk of ischemic stroke, i.e., an absolute risk of 3.1% per year which is comparable to the stroke risk of clinical AF [24]. The currently ongoing ARTESiA trial (NCT01938248) and AFNET-NOAH trial (NCT02618577) are designed to investigate whether treatment with direct-acting oral anticoagulants, reduces the risk of ischemic stroke in patients with device-detected atrial high-rate episodes/subclinical AF.

A population such as in the present study is considered to be at high risk for developing AF and often these new AF episodes occur subclinical. However, in another sub analysis from ASSERT it was shown that the progression of subclinical AF in patients with a pacemaker or defibrillator was strongly associated with hospitalizations for HF [25]. Furthermore, undetected ‘subclinical’ AF may account for a large part of symptom burden, because symptoms of HF and AF are often very similar. The adequate detection of AF in such a population may thus have clinical implications for symptom evaluation in the individual patient and subsequently for further treatment options for better symptom control. The present study confirms a high incidence of (subclinical) AF in patients with HFmrEF and HFpEF and demonstrated a high diagnostic yield of continuous monitoring using an ILR compared with conventional screening. Therefore, this study suggests that the clinical relevance of AF and concomitant atrial myopathy in patients with HFmrEF/HFpEF may be underestimated to date. Future randomized controlled clinical trials are needed to evaluate whether intensive AF screening strategies using an ILR in patients with HFmrEF and HFpEF, or perhaps with a less invasive wearable device that allows continuous monitoring, will indeed lead to better treatments strategies.

## 5. Limitations

The present study has some limitations. First, the sample size is rather modest to perform extensive multivariable analyses and there is risk for type I and type II errors. Second, the primary outcome parameter of VIP-HF was ventricular tachycardia, therefore, tachycardia events were programmed with high priority and AF episodes with lower priority.

## 6. Conclusions

Atrial fibrillation is very common in patients with HF with mildly reduced or preserved ejection fraction, being either present at baseline or diagnosed after the 2-year follow-up in 79% of all patients. AF screening with implantable loop recording led to a much higher diagnostic yield than yearly ECG and two yearly 24 h Holter monitors. The adequate detection of AF in such a population may lead to better symptom evaluation and to further treatment options for better symptom control. Whether the routine use of implantable loop recording for AF detection in these patients will improve outcome needs further study.

## Figures and Tables

**Table 1:** Baseline characteristics of the study population.

	Total *n* = 113	No AF History *n* = 43	AF History *n* = 70	*p*-Value
Age	73 ± 8	70 ± 8	74 ± 8	**0.01**
Sex, female	58 (51%)	18 (42%)	40 (57%)	0.1
Body mass index, kg/m^2^	29.8 ± 5.7	28.7 ± 5.3	30.4 ± 5.9	0.1
**Comorbidities**				
Hypertension	88 (78%)	31 (72%)	57 (81%)	0.2
Coronary artery disease	39 (35%)	19 (44%)	20 (29%)	0.09
Diabetes mellitus	45 (40%)	13 (30%)	32 (46%)	0.1
Renal dysfunction	54 (48%)	19 (44%)	35 (50%)	0.5
Obesity	45 (40%)	16 (43%)	29 (45%)	0.8
Chronic obstructive pulmonary disease	21 (19%)	9 (21%)	12 (17%)	0.6
CHA_2_DS_2_-VASc	5 [4–6]	4 [3–6]	5 [4–6]	0.3
**Echocardiography (*n* = 113)**				
LV ejection fraction, %	54 ± 6	53 ± 7	54 ± 6	0.4
LV ejection fraction ≥50%	85 (75%)	28 (65%)	57 (81%)	0.051
LV mass index, g/m^2^	102 ± 37	116 ± 48	94 ± 26	**0.01**
E/e′	13.1 ± 5.0	12.3 ± 4.6	13.9 ± 5.2	0.2
Mean e′ septal/lateral wall, cm/s	7.5 ± 2.1	6.6 ± 1.7	8.3 ± 2.1	**<0.001**
LA volume index, mL/m^2^	47 ± 16	40 ± 16	50 ± 15	**0.002**
TAPSE. mm	20.4 ± 4.8	21.5 ± 5.6	19.7 ± 4.1	0.06
RV s′, cm/s	11.5 ± 2.7	12.3 ± 2.7	10.8 ± 2.5	**0.01**
TR peak gradient, mmHg	34 ± 11	34 ± 10	34 ± 11	0.9
**Laboratory test (*n* = 113)**				
Creatinin, µmol/L	122 ± 54	125 ± 59	120 ± 51	0.6
eGFR, mL/min/1.73 m^2^	52 ± 21	53 ± 21	52 ± 22	0.7
NT-proBNP, ng/L	1367 [729–2430]	800 [513–1787]	1665 [1065–2553]	**0.003**
**24-Hour Holter (*n* = 112)**				
Mean heart rate	72 ± 13	67 ± 8	74 ± 15	**0.001**
**Cardiac MRI (*n* = 105)**				
**Left ventricle**				
LV ejection fraction, %	53 ± 8	53 ± 9	52 ± 8	0.8
LV end-diastolic volume index, mL/m^2^	89 ± 25	98 ± 27	84 ± 22	**0.004**
LV end-systolic volume index, mL/m^2^	43 ± 17	48 ± 20	41 ± 15	0.06
LV mass index, g/m^2^	57 ± 23	66 ± 28	52 ± 18	**0.005**
LV global longitudinal strain, %	−16.8 ± 5.0	−17.4 ± 4.6	−16.4 ± 5.3	0.3
LV circumferential strain, %	−22.0 ± 6.1	−23.4 ± 6.5	−21.1 ± 5.8	0.07
**Right ventricle**				
RV ejection fraction, %	53 ± 10	58 ± 11	49 ± 8	**<0.001**
RV end-diastolic volume index, mL/m^2^	81 ± 20	78 ± 16	83 ± 22	0.2
RV end-systolic volume index, mL/m^2^	39 ± 15	33 ± 12	43 ± 15	**0.001**
RV global longitudinal strain (%)	−19.4 ± 6.2	−21.3 ± 6.8	−18.2 ± 5.5	**0.01**
Stroke volume/end-systolic volume	1.2 ± 0.6	1.5 ± 0.7	1.0 ± 0.3	**<0.001**
**Atria**				
LA volume index, mL/m^2^	62 ± 22	54 ± 20	68 ± 21	**0.002**
LA emptying fraction, %	29 ± 16	38 ± 15	23 ± 14	**<0.001**
LA reservoir strain, %	13.6 ± 9.2	18.1 ± 10.5	10.8 ± 6.8	**<0.001**
LA conduit strain, % (*n* = 61)	9.9 ± 5.4	10.0 ± 6.1	9.9 ± 3.7	0.9
LA booster strain, % (*n* = 61)	8.0 ± 5.5	8.3 ± 6.2	7.6 ± 4.1	0.7
RA volume index, mL/m^2^	46 ± 22	38 ± 18	51 ± 24	**0.002**
RA emptying fraction, %	28.4 ± 15.9	39 ± 13	21 ± 13	**<0.001**
RA reservoir strain, %	18.5 ± 13.3	24.9 ± 13.2	14.4 ± 11.7	**<0.001**
RA conduit strain, % (*n* = 61)	10.3 ± 6.5	10.7 ± 6.6	9.6 ± 6.3	0.5
RA booster strain, % (*n* = 61)	14.4 ± 9.4	14.3 ± 9.6	14.6 ± 9.4	0.9
**Medication**				
Beta blockers	98 (87%)	34 (79%)	64 (91%)	0.06
ACEi/ARB	72 (64%)	31 (72%)	41 (59%)	0.1
MRA	44 (39%)	18 (42%)	26 (37%)	0.6
Loop diuretics	101 (89%)	38 (88%)	63 (90%)	0.8
Class 1 AAD	0	0	0	
Class 3 AAD	5 (4%)	0	5 (7%)	0.07
Non-dihydropyridine CCB	6 (5%)	3 (7%)	3 (4%)	0.5
Digoxin	23 (20%)	0	23 (33%)	**<0.001**
DOAC/VKA	67 (59%)	0	67 (96%)	**<0.001**

AAD: anti-arrhythmic drugs; ACEi: angiotensin-converting enzyme inhibitor; AF: atrial fibrillation; ARB: angiotensin receptor blocker; CCB: calcium channel blocker; DOAC: direct-acting oral anticoagulant; eGFR: estimated glomerular filtration rate; LA: left atrial; LV: left ventricular; MRA: mineralocorticoid receptor antagonist; MRI: magnetic resonance imaging; NT-proBNP: N-terminal pro brain natriuretic peptide; RA: right atrial; RV: right ventricular; TAPSE: tricuspid annular plane systolic excursion; TR: tricuspid regurgitation; VKA: vitamin K antagonist.

**Table 2:** Cox regression analysis for the association between baseline characteristics with incident atrial fibrillation.

	Univariable Cox Regression Analysis	
	Hazard Ratio [95% CI]	*p*-Value
Age, per year	1.45 [0.90–2.34]	0.1
Male sex	0.95 [0.38–2.38]	0.9
Body mass index, per SD increase	1.35 [0.82–2.22]	0.2
Heart rate, per SD increase	0.85 [0.43–1.71]	0.7
Systolic blood pressure, per SD increase	1.26 [0.89–1.79]	0.2
Diastolic blood pressure, per SD increase	1.04 [0.63–1.71]	0.9
**Comorbidities**		
Hypertension	1.05 [0.37–2.94]	0.9
Diabetes mellitus	0.49 [0.16–1.48]	0.2
Coronary artery disease	1.02 [0.41–2.52]	0.97
CHA_2_DS_2_-VASc	1.09 [0.82–1.46]	0.6
**Echocardiography**		
LV ejection fraction, per SD increase	0.93 [0.62–1.41]	0.7
LV mass index, per SD increase	1.08 [0.79–1.50]	0.6
E/e′, per SD increase	0.85 [0.49–1.50]	0.6
Mean e′ septal/lateral wall, per SD increase	0.84 [0.45–1.59]	0.6
LA volume index, per SD increase	2.10 [1.30–3.42]	**0.003**
TAPSE, per SD increase	1.21 [0.73–1.72]	0.6
RV s′, per SD increase	0.95 [0.59–1.52]	0.8
>mild mitral regurgitation	3.46 [0.97–12.33]	0.056
**Laboratory test**		
NT-proBNP, per Ln increase	1.26 [0.80–1.98]	0.3
eGFR, per Ln increase	0.98 [0.35–2.77]	0.98
**Medication**		
Beta blockers	2.27 [0.52–9.93]	0.3
ACEi/ARB	1.23 [0.45–3.46]	0.7
MRA	0.67 [0.25–1.80]	0.4
Loop diuretics	1.07 [0.25–4.70]	0.9

All continuous variables were transferred to represent 1 standard deviation (SD) change. NT-proBNP and eGFR were log transformed. CI: confidence interval. All other abbreviations as in Table 1.

**Table 3:** Cox regression analysis for the association between cardiac magnetic resonance parameters with incident atrial fibrillation.

	Univariable Cox Regression Analysis	
	Hazard Ratio [95% CI]	*p*-Value
LV end-diastolic volume index, per SD increase	1.10 [0.70–1.72]	0.7
LV end-systolic volume index, per SD increase	1.29 [0.85–1.94]	0.2
LV ejection fraction, per SD increase	0.70 [0.45–1.10]	0.1
LV mass index, per SD increase	0.69 [0.63–1.47]	0.8
RV end-diastolic volume index, per SD increase	1.12 [0.62–2.03]	0.7
RV end-systolic volume index, per SD increase	1.26 [0.72–2.21]	0.4
RV ejection fraction, per SD increase	0.86 [0.56–1.31]	0.4
RV mass index, per SD increase	0.82 [0.52–1.30]	0.4
LA end-systolic volume index, per SD increase	1.94 [1.12–3.34]	**0.017**
LA end-diastolic volume index, per SD increase	2.14 [1.24–3.69]	**0.006**
LA emptying fraction, per SD increase	0.75 [0.43–1.30]	0.3
LA reservoir strain, per SD increase	0.86 [0.54–1.35]	0.5
LA passive strain, per SD increase	0.91 [0.61–1.36]	0.6
LA active strain, per SD increase	0.89 [0.54–1.45]	0.6
RA volume index, per SD increase	1.02 [0.54–1.93]	0.9
RA emptying fraction, per SD increase	0.71 [0.39–1.30]	0.3
RA reservoir strain, per SD increase	0.83 [0.50–1.36]	0.4
RA passive strain, per SD increase	0.95 [0.58–1.55]	0.8
RA active strain, per SD increase	0.60 [0.44–1.31]	0.3
LV global longitudinal strain, per SD increase	0.90 [0.55–1.46]	0.7
LV circumferential strain, per SD increase	0.97 [0.63–1.49]	0.9
RV global longitudinal strain, per SD increase	1.12 [0.73–1.72]	0.6
RV coupling, per SD increase	0.85 [0.53–1.38]	0.5

All continuous variables were transferred to represent 1 standard deviation change. All abbreviations as in Table 1 and Table 2.

## Data Availability

All authors take responsibility for all aspects of the reliability and freedom from bias of the data presented and their discussed interpretation.

## Supplementary Materials

The following supporting information can be downloaded at: https://www.mdpi.com/article/10.3390/jcm12113682/s1, Figure S1: Kaplan-Meier curve for incident AF detected on the ILR; Figure S2: Kaplan-Meier curve for incident AF detected on any (un)planned ECG or 24-hour Holter; Table S1: Baseline characteristics of all study groups.
