# Supplementary material for: Prevalence and Incidence of Atrial Fibrillation in Heart Failure with Mildly Reduced or Preserved Ejection Fraction: (Additive) Value of Implantable Loop Recorders
Source: J Clin Med. 2023 May 26;12(11):3682. doi: 10.3390/jcm12113682 (PMC10253837; doi:10.3390/jcm12113682)
Supplement: Supplementary file 1 [file jcm-12-03682-s001.zip › jcm-2410637-supplementary.pdf]

Supplemental Figure S1: Kaplan-Meier curve for incident AF detected on the ILR.

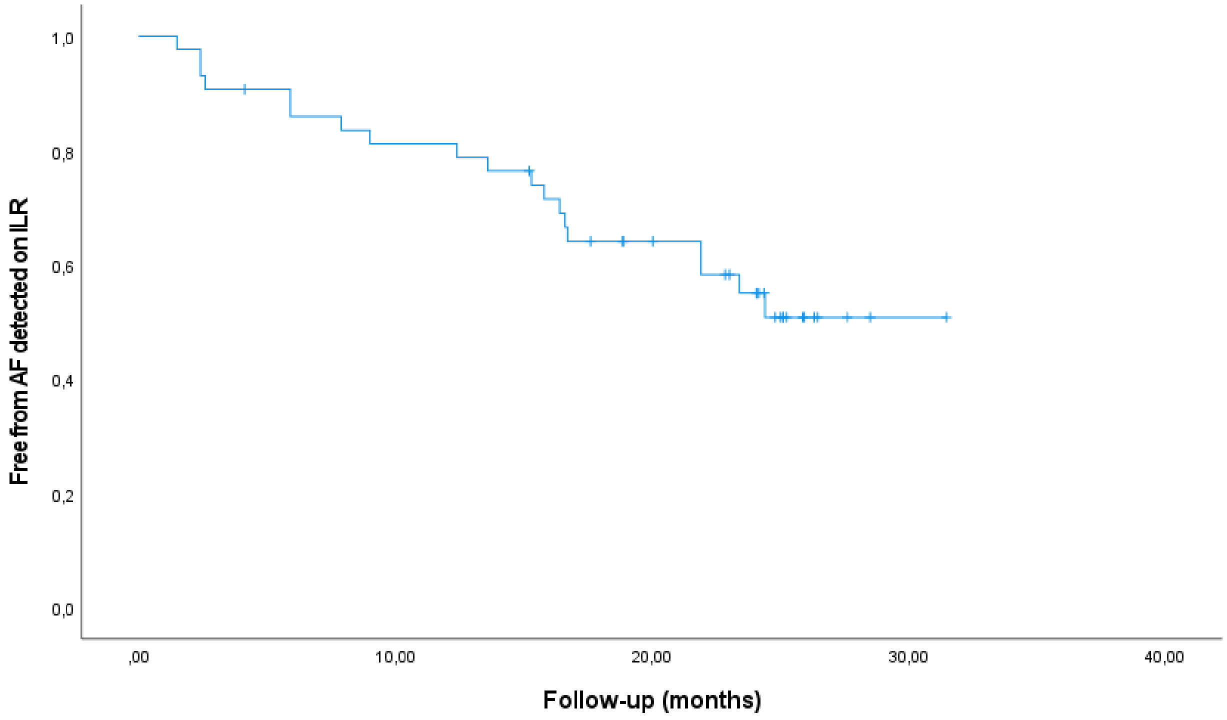

Supplemental Figure S2: Kaplan-Meier curve for incident AF detected on any (un)planned ECG or 24-hour Holter.

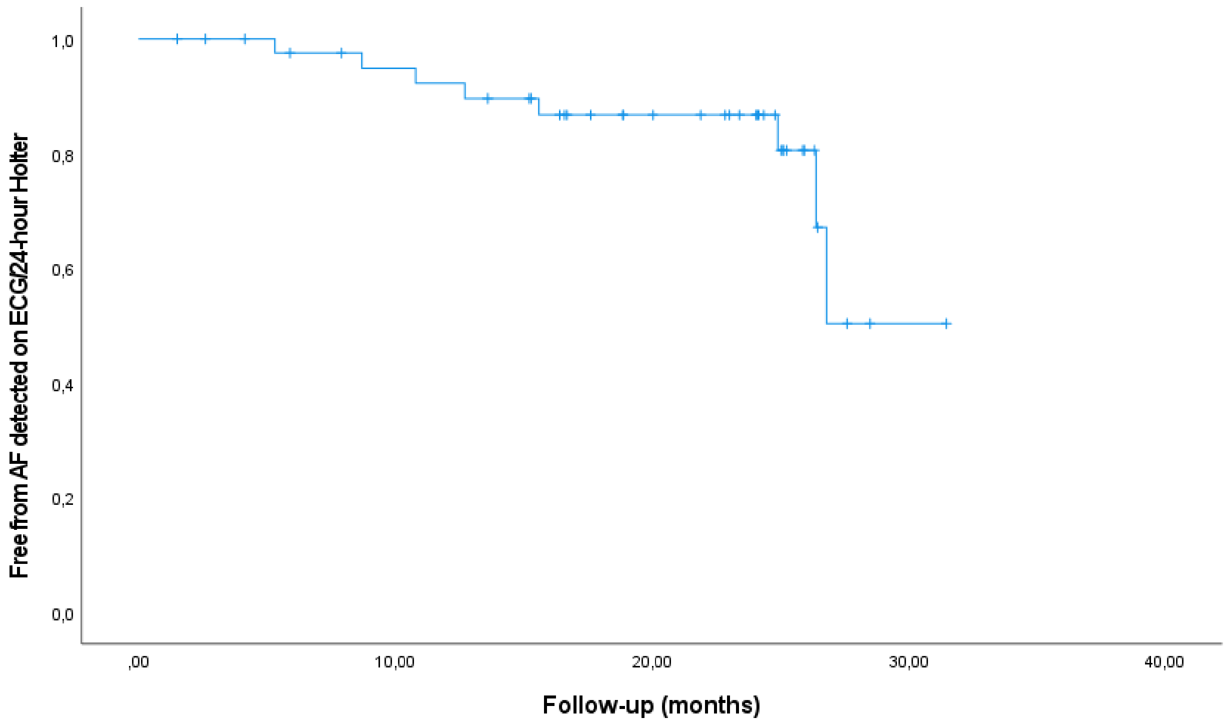

Supplementary Table S1: Baseline characteristics of all study groups

	**Group A: No AF diagnosis n=43**	**Group B: AF with sinus rhythm n=25**	**p-value A vs. B**	**Group C: AF with AF rhythm n=45**	**p-value B vs. C**
Age	70 ± 8	71 ± 9	0.7	76 ± 7	**0.01**
Sex, female	18 (42%)	18 (72%)	**0.02**	22 (49%)	0.06
Body mass index, kg/m^2	28.7 ± 5.3	30.7 ± 5.5	0.2	30.3 ± 6.1	0.8
**AF type**					**<0.001**
Paroxysmal	-	15 (60%)		6 (13%)	
Persistent	-	10 (40%)		8 (18%)	
Permanent	-	0		31 (69%)	
**Comorbidities**					
Hypertension	31 (72%)	18 (72%)	0.99	39 (87%)	0.1
Coronary artery disease	19 (44%)	10 (40%)	0.7	10 (22%)	0.1
Diabetes mellitus	13 (30%)	11 (44%)	0.3	21 (47%)	0.8
Renal dysfunction	19 (44%)	11 (44%)	0.99	24 (53%)	0.5
Obesity	16 (43%)	12 (52%)	0.5	17 (42%)	0.4
COPD	9 (21%)	5 (20%)	0.9	7 (16%)	0.6
**Echocardiography (n=113)**					
LV ejection fraction, %	53 ± 7	55 ± 7	0.3	54 ± 5	0.5
LV ejection fraction ≥50%	28 (65%)	19 (76%)	0.3	38 (84%)	0.4

LV mass index, g/m^2	116 ± 48	91 ± 22	**0.03**	95 ± 27	0.5
E/e’	12.3 ± 4.6	13.7 ± 5.4	0.3	14.0 ± 5.0	0.9
Mean e’ septal/lateral wall, cm/s	6.6 ± 1.7	6.8 ± 1.2	0.6	9.2 ± 2.0	**<0.001**
LA volume index, ml/m^2	40 ± 16	44 ± 14	0.3	54 ± 15	**0.02**
TAPSE, mm	21.5 ± 5.6	20.7 ± 3.9	0.5	19.0 ± 4.1	0.1
RV s’, cm/s	12.3 ± 2.7	11.1 ± 2.4	0.2	10.6 ± 2.5	0.6
TR peak gradient, mmHg	34 ± 10	41 ± 13	0.1	31 ± 10	**0.01**
**Laboratory test (n=113)**					
Creatinin, µmol/L	125 ± 59	107 ± 52	0.2	127 ± 50	0.2
eGFR, ml/min*1.73m^2	53 ± 21	58 ± 23	0.4	48 ± 20	0.08
NT-proBNP, ng/L	840 [512-1905]	648 [636-1591]	0.94	2047 [1345-3438]	**<0.001**
**24-Hour Holter (n=112)**					
Mean heart rate	67 ± 8	68 ± 10	0.9	78 ± 15	**0.002**
**Cardiac MRI (n=105)**					
**Left ventricle**					
LV ejection fraction, %	53 ± 9	56 ± 9	0.2	51 ± 7	**0.02**
LV end-diastolic volume index, ml/m^2	98 ± 27	87 ± 19	0.1	82 ± 23	0.4
LV end-systolic volume index, ml/m^2	48 ± 20	39 ± 13	0.08	41 ± 16	0.6
LV mass index, g/m^2	66 ± 28	48 ± 13	**0.001**	53 ± 20	0.3
LV global longitudinal strain, %	-17.4 ± 4.6	-19.2 ± 5.7	0.2	-15.0 ± 4.5	**0.002**

LV circumferential strain, %	-23.4 ± 6.5	-21.6 ± 6.0	0.3	-20.8 ± 5.7	0.6
**Right ventricle**					
RV ejection fraction, %	58 ± 11	54 ± 7	0.09	47 ± 8	**0.001**
RV ejection fraction <45%	7 (17%)	1 (5%)	0.2	17 (40%)	**0.004**
RV end-diastolic volume index, ml/m^2	78 ± 16	89 ± 23	**0.04**	81 ± 21	0.2
RV end-systolic volume index, ml/m^2	33 ± 12	41 ± 15	**0.03**	44 ± 16	0.5
RV mass index, g/m^2	17 ± 5	17 ± 5	0.8	17 ± 4	0.9
RV global longitudinal strain, %	-21.3 ± 6.8	-20.9 ± 4.7	0.8	-16.9 ± 5.4	**0.006**
Stroke volume / end-systolic volume	1.5 ± 0.7	1.2 ± 0.3	**0.01**	0.9 ± 0.3	**0.001**
**Atria**					
LA end-systolic volume index, ml/m^2	54 ± 20	56 ± 17	0.8	74 ± 21	**0.001**
LA emptying fraction, %	38 ± 15	38 ± 10	0.99	15 ± 9	**<0.001**
LA reservoir strain, %	18.1 ± 10.5	17.5 ± 6.3	0.8	7.5 ± 4.2	**<0.001**
LA conduit strain, % (n=61)	10.0 ± 6.1	9.9 ± 3.7	0.9	-	
LA booster strain, % (n=61)	8.3 ± 6.2	7.6 ± 4.1	0.7	-	
RA end-systolic volume index, ml/m^2	38 ± 18	44 ± 20	0.3	55 ± 24	0.06
RA emptying fraction, %	39 ± 13	34 ± 13	0.2	15 ± 8	**<0.001**
RA reservoir strain, %	24.9 ± 13.2	24.2 ± 12.6	0.8	9.6 ± 7.6	**<0.001**

RA conduit strain, % (n=61)	10.7 ± 6.6	9.6 ± 6.3	0.5	-	
RA booster strain, % (n=61)	14.3 ± 9.6	14.6 ± 9.4	0.9	-	
**Medication**					
Beta blockers	34 (79%)	22 (88%)	0.4	42 (93%)	0.4
ACEi/ARB	31 (72%)	12 (48%)	**0.047**	29 (64%)	0.2
MRA	18 (42%)	13 (52%)	0.4	13 (29%)	0.06
Loop diuretics	38 (88%)	20 (80%)	0.3	43 (96%)	**0.04**
Class 1 AAD	0	0		0	
Class 3 AAD	0	2 (8%)	0.06	3 (7%)	0.8
Non-dihydropyridine CCB	3 (7%)	1 (4%)	0.6	2 (4%)	0.9
Digoxin	0	3 (12%)	**0.02**	20 (44%)	**0.006**
DOAC/VKA	0	23 (92%)	**<0.001**	44 (98%)	0.2

*p<0.05 (Group A vs. Group C). AAD anti-arrhythmic drugs; ACEi angiotensin-converting enzyme inhibitor; AF atrial fibrillation; ARB angiotensin receptor blocker; CCB calcium channel blocker; DOAC direct-acting oral anticoagulant; eGFR estimated glomerular filtration rate; LA left atrial; LV left ventricular; MRA mineralocorticoid receptor antagonist; MRI magnetic resonance imaging; NT-proBNP N-terminal pro brain natriuretic peptide; RA right atrial; RV right ventricular; TAPSE tricuspid annular plane systolic excursion; TR tricuspid regurgitation; VKA vitamin K antagonist.
